# Rehabilitation approaches to anterior knee pain among runners: A scoping review

**DOI:** 10.4102/sajp.v76i1.1342

**Published:** 2020-01-27

**Authors:** Siyabonga H. Kunene, Nomathemba P. Taukobong, Serela Ramklass

**Affiliations:** 1 Department of Physiotherapy, Faculty of Health Sciences, University of the Witwatersrand, Johannesburg, South Africa; 2 Institutional Planning Department, Faculty of Administration and Support, Sefako Makgatho Health Sciences University, Pretoria, South Africa; 3 School of Clinical Medicine, Faculty of Health Sciences, University of KwaZulu-Natal, Durban, South Africa

**Keywords:** anterior knee pain, rehabilitation strategies, runners, scoping review, education, gait re-education, exercise, foot orthoses and multimodal rehabilitation

## Abstract

**Background:**

Many athletes complain of anterior knee pain (AKP) which is the most common clinical problem, with a prevalence of 15% – 45%, posing a threat to their quality of life. Owing to a lack of consensus among clinicians and researchers, the causes and management of AKP remain controversial.

**Objectives:**

The aim of this study was to map the range of non-surgical and non-pharmaceutical rehabilitation approaches to AKP among runners.

**Method:**

A scoping review was conducted in five stages: (1) defining the research question, (2) identifying relevant studies, (3) selecting a topic, (4) charting and collecting data and (5) summarising and reporting the results. Included in the study were English original articles on AKP rehabilitation strategies for runners prior to November 2019. Six electronic databases were searched: EBSCOHOST, CINAHL, SPORTDISCUS, PUBMED, COCHRANE and SCOPUS.

**Results:**

Thirteen out of 1334 articles met the inclusion criteria. Two reviewers independently participated in the screening and extraction of articles. The identified articles included four randomised controlled trials, one systematic review, four observational studies, one cohort study, two case studies and one quasi-experimental study. The following rehabilitation strategies were found to be useful: education, gait re-education, exercise, foot orthoses and multimodal rehabilitation.

**Conclusion:**

This study provided a range of rehabilitation strategies that were found useful in the rehabilitation of AKP. More comprehensive intervention studies are needed to address all physical and non-physical features of AKP.

**Clinical implications:**

The outcomes of this study make explicit the usefulness of the identified rehabilitation strategies among runners with AKP. These will guide clinicians in the development of rehabilitation programmes for runners.

## Introduction

Anterior knee pain (AKP) is felt at the front, centre and around the patellofemoral joint (Brukner & Khan 2017). Being a common clinical presentation among runners, it presents a threat to their quality of life (QoL). It has a global prevalence of 15% – 45% (Cook et al. 2010), with females and a younger population showing a greater prevalence (Boling et al. 2010; Luhmann et al. 2008; Saxena & Haddad 2003). Owing to the lack of consensus among clinicians and researchers, its causes and management remain controversial (Brukner & Khan 2017; Hreljac 2005; Juhn 1999). Several factors contribute to AKP, and these include both internal and external factors. Internal factors include structural dysfunctions of the body, such as muscle force imbalances. External factors, to name but a few, include running equipment, the environment, training load, running method and gait. All of these factors make AKP a challenging condition for clinicians to manage. Anterior knee pain requires adequate assessment and screening of possible risk factors to come up with a relevant and comprehensive treatment and rehabilitation programme. The causes of AKP tend to differ from athlete to athlete, depending on the structure of the affected knee, thus making it important to assess and identify the causes of AKP (Brukner & Khan 2017).

Numerous structures of the knee can be susceptible to overload and overuse during activities involving the knee. The soft tissues around the knee, for example, the infrapatellar fat pad and the lateral retinaculum, are considerable sources of pain among patients (Brukner & Khan 2017).

Poor lower limb alignment, including an increased Q-angle, subtalar pronation or *pes planus*, can lead to AKP (Dixit et al. 2007). When the knee is flexed, the patella moves medially in relation to the knee, so that it can sit properly in the intercondylar notch until a knee-joint angle of 130° is achieved. After this range has been attained, the patella will immediately slide back laterally (Brukner & Khan 2017).

The quadriceps muscles, particularly the components of the *vastus medialis* oblique (VMO) and the *vastus lateralis* oblique (VLO), are the main forces that control the movement of the patella during knee action (Brukner & Khan 2017). As opposed to the medial knee structures, the lateral soft tissue of the knee structures tends to become tighter and much stronger. This will then cause an imbalance between the forces that act upon the patella and may result in poor movement coordination of the knee. Therefore, if the VMO is weak, the VLO is too tight and excessive load is put on the knee during running, pain may result on account of the irritation or excessive pressure exerted beneath the patella and the other associated structures (Brukner & Khan 2017).

The non-operative physical management of athletes with AKP includes correcting the abnormal patellofemoral kinematics and the alignment of the knee joint. This correction can be done by improving the strength of the VMO and its activation and timing, and the flexibility of the shortened soft tissue structures (e.g. VLO), and by correcting the patellar alignment by applying strapping and knee guards (Dutton, Khadavi & Fredericson 2014). It is important to also address the biomechanical problems of the lower limb extremities that include weakness in the hip, poor foot position and gait (Dutton et al. 2014).

There is limited evidence on the use of some physical treatments for AKP. However, physiotherapy has been shown to improve short-term pain and function among athletes (Crossley et al. 2001). Podiatry also plays an important role in pain management and the correction of biomechanical problems by prescribing appropriate shoes and orthosis for runners with foot alignment problems (Saxena & Haddad 2003). It has been shown that increased foot pronation can be a risk factor for AKP (Halabchi, Mazaheri & Seif-Barghi 2013; Kunene, Ramklass & Taukobong 2018a).

Analgesics (non-steroidal anti-inflammatory drugs), bracing (braces, sleeves and straps) and patellar taping are also considered as part of the treatment for AKP (Dixit et al. 2007). Operative treatment for patients with AKP may be necessary, especially if non-operative treatment is ineffective (Brukner & Khan 2017).

To be effective in the treatment and rehabilitation of knee conditions, a team work approach among professionals has been shown to result in successful outcomes in rehabilitation (Strasser, Uomoto & Smits 2008). In professional sports, depending on the rehabilitation needs and costs involved, interdisciplinary rehabilitation team members generally include a variety of health care practitioners, such as sports physicians, physiotherapists, psychologists, nutritionists or dieticians, biokineticists, massage therapists, podiatrists and other professionals. In my experience as a clinician, personal trainers and coaches also form part of the rehabilitation team. Each member of the team uses his/her knowledge and skills appropriately in a particular stage in the management of the injury. Communication among such members is always crucial to enhance the continuity and the quality of care of the athletes. Unfortunately, sporting clubs in poorly resourced communities do not have the luxury of having access to a full rehabilitation service. This may result in inadequacies in the rehabilitation of injuries.

Evidence of rehabilitation strategies that are being or have been used in the treatment of runners with AKP is limited. Therefore, the aim of this review was to map the range of non-surgical and non-pharmaceutical rehabilitation approaches to AKP among runners.

### Review methodology

A preparatory assessment of the possible scope and size of the available research literature was conducted. The authors identified the inherent features and the depth of the research evidence at hand on the strategies used to rehabilitate runners with AKP. Our review used the methodological frameworks as outlined by Arksey and O’Malley (2005) and Levac, Colquhoun and O’Brien (2010). The process included five stages. They are listed in sequence as follows: (1) defining the research question, (2) identifying the relevant studies, (3) selecting the main theme for this study, (4) charting and collecting the data and (5) summarising and reporting the results.

### Defining of research question

Firstly, a research question was defined by the first author in consultation with three experts, who were sports physiotherapists with at least 10 years of clinical experience and 5 years of research experience. The research question was refined as follows: what are the current non-pharmacological and non-surgical rehabilitation approaches to AKP among runners?

### Search strategy

After the research question had been defined, a comprehensive process of identifying and selecting relevant studies available from the various databases was conducted by two independent researchers. An Internet search strategy was developed with the assistance of a librarian, who had a vast knowledge of and was experienced in the methods of searching for literature sources.

The search was performed in three steps as follows. Step 1: this included a search on MEDLINE (PubMed) and CINAHL. It was then followed by an analysis of the text words in both the titles and abstracts of each of the selected articles. The index terms for each article were also analysed at this stage. The MESH terms that were used initially included ‘patellofemoral pain syndrome’ OR ‘anterior knee pain’ AND runner. Step 2: this step included a search that was conducted on the basis of the keywords and index terms in the following six databases: EBSCOHOST, CINAHL, SPORTDISCUS, PUBMED, COCHRANE and SCOPUS.

Step 3: this step included a search for additional studies that were based on the use of the reference lists of the articles that had already been identified.

This review included original research articles that had initially been peer-reviewed prior to November 2019. Only human subjects (runners) were considered, but all of the studies looking at strategies for the rehabilitation of runners with AKP (only non-pharmacological and no-surgical studies) were accepted as relevant to the study. Articles written in languages other than English were excluded.

### Data extraction and analysis

The selection of relevant studies was then followed by a process involving the extraction and charting of the data. The references to the electronic articles were downloaded onto the EndNote programme. All duplicates and articles that did not meet the inclusion criteria were removed. To avoid bias, the first author and his assistant then screened the identified articles independently of one another. The full-text versions of the articles that were relevant were then downloaded for further evaluation. The following were evaluated from the extracted articles: the name of the authors, publication year, study location, study design, aims/purpose of the study, study population and sample size, study methods, outcome of the study and limitations, if any.

Experts reviewed the extracted information to confirm whether all of the aspects had been executed accurately. Lastly, summaries were made of our findings in the form of a table.

### Ethical consideration

This article followed all ethical standards for research without direct contact with human or animal subjects.

## Results

### Description of studies

**FIGURE 1:**
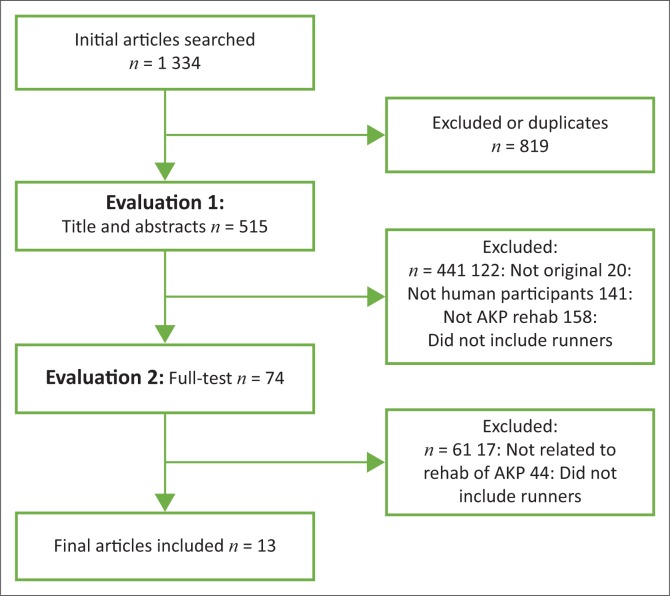
Article evaluation flow chart detailing the search process.

The initial search process yielded 1334 articles (Figure 1). Five hundred and fifteen articles were included for Phase 1 of the evaluation process after duplicates, if any, had been removed. After removing the articles that did not meet the criteria in terms of titles and abstracts, 74 full-text articles were finally extracted and included for further evaluation. Out of the 74, only 13 met the inclusion criteria after the final evaluation process (full-text evaluation).

Details of the 13 articles included for final analysis and discussion are shown in Table 1. The studies used a variety of study designs that included four randomised controlled trials (RCTs) (Bonaccia et al. 2018; Esculier et al. 2018; Roper et al. 2016; Shih, Wen & Chen 2011), two case studies (Cheung & Davis 2011; Willy & Davis 2013), one cohort study (Ferber, Kendall & Farr 2011), one quasi-experimental study (Esculier, Bouyer & Roy 2016), one systematic review (Neal et al. 2016) and four prospective observational studies (Boldt et al. 2013; Noehren, Scholz & Davis 2011; Rodrigues et al. 2013; Willy, Scholz & Davis 2012).

**TABLE 1:** Summary of studies included in the study (*n* = 13).

Author(s)	Title	Design	Setting	Participants	Findings	Conclusion
Boldt et al. (2013)	Effects of medially wedged foot orthoses on knee and hip-joint running mechanics in females with and without patellofemoral pain syndrome	Observational study	The United States	Twenty female runners with and without AKP aged 18–35 years, who ran at least 10 miles/week and reported their activity level as greater than or equal to five out of 10 on the Tegner activity scale	Knee abduction moment increased (*p* = 0.03) and hip adduction excursion decreased (*p* < 0.01) when using medially wedged foot orthoses. The effect that the orthoses exercised on the knee and hip mechanics was minimal	Medially wedged foot orthoses are minimally effective in correcting knee and hip biomechanics
Bonaccia et al. (2018)	Gait retraining versus foot orthoses for patellofemoral pain: a pilot randomised clinical trial	A pilot randomised clinical trial	Australia	Sixteen runners aged 18–40 years with AKP, which was non-traumatic and did not last longer than 6 weeks and was provoked by activity; worst pain over the previous week; running at least 10 km/week	A 6-week gait-retraining programme has a clinically meaningful effect on runners with AKP when compared to an evidence-based treatment of foot orthoses	Gait-retraining programme more effective as opposed to programmes using foot orthotics
Cheung and Davis (2011)	Landing pattern modification to improve patellofemoral pain in runners: a case series	Case series	China	Three female runners aged 26–32 years who ran 20–30 km/week, suffered unilateral patellofemoral pain and presented with a rearfoot strike pattern	A change in landing pattern from a rearfoot to a non-rearfoot strike pattern resulted in reduced vertical impact peak and rates of load, AKP symptoms, functional limitations. Improved running performance was also noted	Non-rearfoot strike effective in reducing vertical impact peak and rates of load, AKP symptoms and functional limitations. Improved running performance noted
Esculier et al. (2016)	The effects of a multimodal rehabilitation programme on symptoms and ground-reaction forces in runners with patellofemoral pain syndrome	Pre- to post- and quasi-experimental	Canada	Twenty-one runners aged 18–45 years, with AKP, running at least 15 km/week, and reporting symptoms for at least 3 months	The intervention significantly reduced pain and limited function among runners (*p* < 0.001) and instantaneous vertical loading rate (*p* = 0.002)	Multimodal rehabilitation programme effective to reduce pain and limited function
Esculier et al. (2018)	Is combining gait retraining or an exercise programme with education better than education alone in treating runners with patellofemoral pain? A randomised clinical trial	Single-blind randomised clinical trial	Canada	Sixty-nine runners aged 18–45 years, with a minimal weekly running distance of 15 km, and presenting with AKP for at least 3 months; experiencing minimum pain levels of 3/10 (VAS) and scoring a maximum of 85/100 on KOS-ADLS	Education alone was found to be as efficient as education combined with exercises or gait retraining in reducing the symptoms and functional limitations of runners with PFP	Education is efficient in reducing AKP symptoms and functional limitations
Ferber et al. (2011)	Changes in knee biomechanics after a hip-abductor strengthening protocol for runners with patellofemoral pain syndrome	Cohort study	Canada	Twenty-five active recreational runners running at least 30 min/day, 3 days/week	A 3-week hip-abductor muscle-strengthening protocol led to increased muscle strength, reduced pain and stride-to-stride knee-joint variability (*p* < 0.05). No change was noticed in the peak *genu valgus* angle	Exercises effective to improve abductor strength and knee pain and stride-to-stride knee-joint variability
Neal et al. (2016)	Runners with patellofemoral pain have altered biomechanics which targeted interventions can modify: a systematic review and meta-analysis	Systematic review and meta-analysis	The United Kingdom	Twenty-eight studies investigated AKP in a running cohort in a prospective, case control or intervention study. A mixed-sex cohort group	Gait retraining significantly reduced peak hip adduction. Minimal evidence indicated that both running retraining and proximal strengthening exercises were favourable for reducing pain and improving function	Gait retraining reduced peak hip adduction
Noehren et al. (2011)	The effect of real-time gait retraining on hip kinematics, pain and function in subjects with patellofemoral pain syndrome	Observational study	The United States	Ten female recreational runners, aged 18–45 years, with AKP for at least 2 months	After gait retraining, there was a significant reduction in hip adduction, contralateral pelvic drop, pain and improvement of function (*p* ≤ 0.05). Hip internal rotation diminished with no significant difference. Noted changes were maintained for a month	Gait retraining was effective to reduce hip adduction, contralateral pelvic drop, pain. Gait retraining was also found effective to improve function
Rodrigues et al. (2013)	Medially posted insoles consistently influence foot pronation in runners with and without anterior knee pain	Observational study	The United States	Sixteen asymptomatic (seven males, nine females) and 17 runners with AKP (four males, 13 females)	Insoles reduced peak eversion, peak eversion velocity and range of motion (*p* < 0.01) but had only a limited influence on knee or tibia transverse plane kinematics. There was also a change in peak tibial internal rotation (*p* = 0.08) and transverse plane knee ROM (*p* < 0.01) and peak knee internal rotation (*p* = 0.06)	Medial insoles reduced peak version, peak velocity and range of motion
Roper et al. (2016)	The effects of gait retraining in runners with patellofemoral pain: a randomised trial	A randomised trial	The United States	Twenty-one recreational runners aged 21–34 years, with a running volume of 26–33 km/week	Running with a forefoot strike pattern led to reduced knee pain, knee abduction, ankle plantar flexion, post-retraining 1 month later (*p* < 0.05)	Forefoot running method effective to reduce knee pain, knee adduction and ankle plantar flexion
Shih et al. (2011)	Application of wedged foot orthosis effectively reduces pain in runners with pronated foot: a randomised clinical study	Randomised clinical trial	China	Twenty-four long-distance runners (18 males and six females) with pronated foot	Knee and foot pain were reduced after wearing foot orthosis in the treatment group (*p* = 0.04)	Foot orthosis reduced knee and foot pain
Willy and Davis (2013)	Varied response to mirror gait retraining of *gluteus medius* control, hip kinematics, pain, and function in two female runners with patellofemoral pain	Case report	The United States	Two college-aged female runners with chronic anterior knee pain	Both cases showed positive changes in knee pain, function, hip mechanics, *gluteus medius* control and hip abduction strength. Cases showed varied responses in terms of skill transfer of improved hip mechanics and *gluteus medius* activation to the task of step ascent	Mirror gait retraining effective to reduce knee pain, and to improve function, hip mechanics and strength
Willy et al. (2012)	Mirror gait retraining for the treatment of patellofemoral pain in female runners	Observational study	The United States	Ten females aged 18–40 years, running at least 10 km/week	There was a reduced peak of hip adduction, contralateral pelvic drop and hip abduction moment, and skills transfer to single leg squatting, and step descent was noted (*p* < 0.05). Pain was reduced and function was improved (*p* < 0.05). Changes were maintained for 3 months	Mirror gait retraining effective to reduce knee pain and to improve hip control and function

AKP, anterior knee pain; VAS, visual analogue scale; KOS-ADLS, knee outcome survey activities of daily living scale; ROM, range of motion.

These studies were conducted in various places, most of them in the United States (Boldt et al. 2013; Noehren et al. 2011; Rodrigues et al. 2013; Roper et al. 2016; Willy & Davis 2013; Willy et al. 2012), followed by Canada (Esculier et al. 2016, 2018; Ferber et al. 2011), China (Cheung & Davis 2011; Shih et al. 2011), Australia (Bonaccia et al. 2018) and the United Kingdom (Neal et al. 2016). All studies included runners with or without AKP.

### Study outcomes

All the studies reviewed focussed on the non-surgical and non-pharmacological interventions for the rehabilitation of runners with AKP.

#### Education

According to the study by Esculier et al. (2018), health education of runners about their condition (AKP) should be considered as part of the rehabilitation programme. Their programme included teaching runners about AKP symptoms and the management of training loads. They found that education alone was as efficient as the combined programme of education and exercises or gait retraining in reducing AKP symptoms and functional limitations. Their study also showed the important role education plays in the rehabilitation of runners with AKP. In their study, education was included as part of the multimodal rehabilitation programme, which was found to be effective in reducing pain and increasing function in runners with AKP.

#### Gait retraining

Six articles described the effects of gait retraining on various problems associated with AKP among runners. Willy et al. (2012) considered the effect of mirror gait retraining on AKP among female runners.

They found that the gait-retraining programme was effective using a full-length mirror. This programme reduced the participant’s pain, and improved function and body mechanics. The improvements suggest the likelihood of long-term benefits. These improvements were found to last for 3 months. In a case report by Willy and Davis (2013), 2 weeks of mirror gait retraining was also found to be effective in addressing pain, function, hip mechanics, *gluteus medius* control and hip-abductor strength.

Roper et al. (2016) conducted a randomised control trial (RCT) among male and female recreational runners, evaluating the impact of gait retraining among the running population experiencing the problem of AKP. They found that changing the running pattern from rearfoot strike to forefoot strike improved the runners’ symptoms. This method reduced knee pain and improved the biomechanics of the knee and the ankle. They concluded that the forefoot-strike running-gait pattern should be considered as part of the rehabilitation of runners with AKP.

Cheung and Davis (2011) also found similar results that support the effects of the forefoot landing pattern. In their case series report, they found that the changed landing pattern from rearfoot to forefoot landing reduced knee pain and the associated functional limitations among three participants. Owing to the gait-retraining programme, one of the runners showed an improvement in running performance.

Noehren et al. (2011) conducted a study among 10 runners with AKP to describe the benefits of a real-time gait retraining programme on three components. These included reduced knee pain, improved function and hip kinetics. A pilot RCT by Bonaccia et al. (2018) showed that a 6-week gait retraining programme was more effective in reducing knee pain and improving function than foot orthoses.

Neal et al. (2016) also reported that running retraining is effective in reducing the associated problems in AKP. Running-gait retraining reduces peak hip adduction among runners with AKP. The authors concluded that the correction of biomechanical problems among runners with AKP can mitigate the symptoms associated with AKP.

#### Exercise

Ferber et al. (2011) reported the effectiveness of a hip-abductor-strengthening programme on reducing knee pain and improving the strength of the hip-abductor muscle, and stride-to-stride knee-joint variability among runners with AKP. Their rehabilitation protocol consisted of two hip abduction exercises using a resistant-A-band in different directions. Ferber et al. (2011) suggested that a hip-abductor-strengthening programme be considered when rehabilitating runners with AKP.

In a systematic review by Neal et al. (2016), proximal strengthening exercises were shown to be beneficial (with limited evidence) and to lead to diminished knee pain, improvements in muscle strength and function.

No evidence was found with regard to the proximal strengthening exercise programme altering internal rotation, hip abduction, rearfoot eversion, knee abduction or *genu valgus*.

#### Foot orthosis

Shih et al. (2011) considered the effects of the application of foot orthosis using wedges to reduce knee pain for runners with issues of increased foot pronation. They found that the insertion of foot orthotics reduced pain, showing that medially wedged insoles are an effective way to manage AKP and foot-related symptoms. Boldt et al. (2013), in their study on 20 female runners with and without AKP, examined the benefits of using medially wedged foot orthotics on improving the running mechanics of the knee and the hip.

They found that a full-length insole yielded minor positive effects on knee and hip mechanics during running. A conclusion was therefore drawn that the reduced pain among runners with AKP, which had been reported previously, may not have been as a result of changes in joint mechanics arising from the prescription of these types of orthotics.

#### Multimodal rehabilitation

One pre- to post- and quasi-experimental study by Esculier et al. (2016) examined a multimodal rehabilitation programme on 21 runners with AKP. Their intervention involved an 8-week programme, which included the strengthening of the lower limb and core muscles, exercises to improve motor control and advice on running gait, symptom management and a training regimen. They found an improvement in pain and function among runners with AKP but no improvement in lower limb strength was observed.

## Discussion

This review aimed at mapping the range of rehabilitation approaches (non-surgical and non-pharmaceutical) to AKP among runners. Only 13 published articles were reviewed. Five themes emerged from the reviewed articles: education, gait re-education, exercise, foot orthosis and multimodal rehabilitation. Most studies that were included were conducted in developed countries; no studies that had been conducted in Africa or South Africa could be found.

In their previous studies, Kunene et al. (2018a) and Kunene, Ramklass and Taukobong (2018a, 2018b) identified a need for the development of a rehabilitation programme for AKP among runners in South Africa, especially in communities with limited resources. In the studies that they conducted, a large number of runners with AKP and a variety of contributing factors were found, which put these runners at risk of this type of overuse injury. They also found a negative impact of AKP on QoL among many of these runners. This review gave an insight into the rehabilitation approaches available to rehabilitate runners with AKP.

Education is one of the key strategies that are used in the general rehabilitation of patients. This strategy should be considered to empower runners with the relevant knowledge and skills to prevent or rehabilitate runners with AKP. As suggested by Esculier et al. (2018), runners should be educated regarding their symptoms and how to manage their training load. It is crucial that runners have an understanding of their condition and its management so that they are able to take charge and fully participate in their rehabilitation process.

Kunene et al. (2019) found that training load is one of the external risk factors among runners in communities with limited resources. Both overtraining and undertraining could contribute to AKP. Therefore, load of training is a critical factor that should be considered when designing prevention and rehabilitation programmes for runners with AKP. According to Esculier et al. (2018), runners with AKP are required to modify their training programmes and should be guided by their symptoms, especially pain. They may need to reduce the frequency level of their weekly running, the speed and duration of their training and avoid running on downhill routes. Within 60 min after training, they should monitor their pain and ensure that it returns to the level it was at before training. Their running distance should be gradually increased, while their symptoms should be monitored before increasing their speed and their level of inclination.

Poor hip control has been identified as another contributing factor to AKP among runners (Halabchi et al. 2013; Kunene et al. 2018a). The drop of the pelvis, hip abduction and internal rotation are related problems that can cause an augmented Q-angle, which may lead to the patellofemoral joint being overloaded on the lateral aspect. According to Dierks et al. (2008), a weak hip-abductor muscle condition among runners with AKP was found to be associated with increased hip abduction during running. Poor hip biomechanics is a serious problem that is caused by poor control of the hip muscle, which includes mainly the weak *gluteus medius* and *maximus* muscles. Therefore, proximal strengthing of the lower limb muscles is necessary to improve hip control (Ferber et al. 2011). There is, however, little evidence to the effect that hip-strengthening exercises alone are effective in improving hip control and in dealing with its associated problems (Willy & Davis 2011). Therefore, a holistic approach is necessary.

Gait training is highly effective in the rehabilitation of AKP among runners (Bonaccia et al. 2018; Cheung & Davis 2011; Neal et al. 2016; Noehren et al. 2011; Roper et al. 2016; Willy & Davis 2013; Willy et al. 2012). According to Willy et al. (2012) and Willy and Davis (2013), mirror gait training improves the hip biomechanics and knee function, and reduces the pain levels.

Noehren et al. (2011) indicated that real-time gait retraining is also effective in terms of its effects on the three components: hip kinematics, knee function and pain. According to Bonaccia et al. (2018), gait retraining has been found to be more effective than the use of foot orthotics. Evidence suggests that foot-landing modification is required to mitigate the problem of AKP in runners. A change from rearfoot- to forefoot running strike is necessary to reduce the vertical impact peak, the rate of leading, pain and functional limitations (Cheung & Davis 2011; Roper et al. 2016). Modifications to foot landing may also improve running performance.

Proximal strengthening programmes are among the more effective ways to strengthen weak muscles, including *gluteus medius* and *maximus* and *vestus medialis* oblique muscles, which have generally been found to be weak in runners with AKP. The weakness of these muscles has been classified as a risk factor for AKP (Halabchi et al. 2013; Kunene et al. 2018a). Findings from this review suggest that a 3-week hip-abductor-muscle-strengthning protocol is helpful in improving muscle strength, eliminating pain and limiting stride-to-stride knee-joint variability among runners with AKP (Ferber et al. 2011). Neal et al. (2016) reported that a proximal strength exercise programme brings with it benefits for addressing pain and dysfunction among runners with AKP. Therefore, proximal strength exercises should be included in the overall rehabilitation programme for runners with AKP. They can also be included as part of AKP prevention programmes.

Exercises also play a role in addressing other problems that may lead to AKP (e.g. a weak core and a tight iliotibial band, hamstring, psoas, gastrocnemius, hip internal rotators, adductor *longus* and *brevis*). Further studies are required to provide more evidence on the effectiveness of exercise programmes on these other risk factors.

Increased foot pronation is one of the problems that runners with AKP have to face, and it has been identified as one of the intrinsic modifiable risk factors (Halabchi et al. 2013; Kunene et al. 2018a). Runners tend to present with increased compensatory pronation movements of the subtalar and/or midtarsal joint when bearing weight during running (Halabchi et al. 2013). This causes biomechanical problems at the patellofemoral joint, which result in a runner sustaining an AKP injury. This review has shown that foot orthosis is a useful intervention in the rehabilitation of AKP for runners with increased foot pronation. A medially wedged insole is effective in preventing or reducing knee pain or foot symptoms, foot eversion and eversion velocity amoung runners with pronated foot (Boldt et al. 2013; Rodrigues et al. 2013; Shih et al. 2011).

However, foot insoles have a very limited effect on the kinematics of the transverse plane of the tibia or the knee, meaning that they have more of an effect on other variables (Rodrigues et al. 2013).

The outcomes of this study suggest that foot orthoses are considered to be one of the strategies to prevent and rehabilitate AKP among runners with increased foot pronation problems. Therefore, a careful foot assessment and presciption of the appropriate shoes and accessories (e.g. insoles) should be carried out.

Kunene et al. (2018b) recommend in their studies that a comprehensive rehabilitation programme should encompass the physical and non-physical components of AKP to improve the QoL for runners. For this to be achieved, a multimodal rehabilitation programme may need to be designed for runners. Esculier et al. (2016) described the effect of a multimodal programmme on the symptoms and ground-reaction force that do not feature in all of the associated problems of AKP. Their multimodal intervension has been successful in mitigating pain and improving function among runners with AKP. Their programme comprises 8 weeks of exercises to strengthen the lower limbs, core strength and motor control. Furthermore, patients should be educated on running gait and symptom management.

Collins et al. (2012) are in support of a multimodal approach of AKP rehabilitation. In this study, the authors found that exercise, patella taping, foot orthoses and acupuncture are more effective in managing AKP if implemented as part of the multimodal physiotherapy programme.

Therefore, a combination of rehabilitation modalities is a strategy that can be considered to address the diverse AKP problems among runners. Clinicians should carefully select modalities that address the specific needs of the runners according to their clients’ assessment/screening outcomes. Runners need to be screened on a regular basis in order for their clinicians to come up with relevant prevention and rehabilitation programmes for AKP. It is crucial that runners be educated about AKP, it being the most common overuse injury. If runners are empowered, they would be more likely to take ownership of their training load management and adhere to their prevention and rehabilitation programmes without seeking the services of a health care provider (e.g. a physiotherapist).

The goals of a rehabilitation programme for AKP should include three phases that would be based on the progress of the patient (Werner 2014). The early phase addresses the impairments such as, among others, pain and swelling, muscle imbalances (tightness and flexibility), gait abnormalities and patellofemoral joint-loading issues. The next phase should aim at correcting postural problems and lower extremity coordination issues, increase the strength of the quadriceps and hip muscles, restore knee function and allow for the patient’s gradual return to regular activity. The last phase should focus more on functional exercises. It takes a committed and dedicated runner to adhere to the rehabilitation programme.

## Conclusion

Anterior knee pain is a challenge to many runners. It is a condition whereby muscle factors require a multidimensional, yet individualised management approach. This study has identified a range of rehabilitation strategies that have been found to be useful in attempting to prevent this problem and to rehabilitate runners. Such strategies include the education of the patient, gait retraining, exercises, the use of foot orthotics and multimodal rehabilitation. These strategies are good, but they may need to be modified to suit various contexts. Some communities, especially low socio-economic communities, have limited resources that can make it difficult to access and afford some of the rehabilitation services.

Considering the diversity of AKP among runners, this study could not provide comprehensive rehabilitation strategies that address all of the needs, both physical and non-physical, of runners with AKP. Therefore, further interventional studies should be undertaken to address the other needs that runners with AKP have and that they might face as they enter the rehabilitatory phase of their journey back towards a pain-free and mobile life.
